# Canine parvovirus NS1 induces host translation shutoff by reducing mTOR phosphorylation

**DOI:** 10.1128/jvi.01463-24

**Published:** 2024-11-27

**Authors:** Xinrui Wang, Xiangqi Hao, Yaning Zhao, Xiangyu Xiao, Shoujun Li, Pei Zhou

**Affiliations:** 1 Guangdong Technological Engineering Research Center for Pets, College of Veterinary Medicine, South China Agricultural University 554665 , Guangzhou, Guangdong, China; Cornell University Baker Institute for Animal Health, Ithaca, New York, USA

**Keywords:** Canine parvovirus type 2 (CPV-2), nonstructural protein 1 (NS1), host translation shutoff, mTOR

## Abstract

Canine parvovirus type 2 (CPV-2) is a member of the Parvoviridae family, characterized by its small, non-enveloped virions containing a linear single-stranded DNA genome of approximately 5 kb. Parvoviruses entirely reliant on the host cell’s division machinery for replication. In this study, we demonstrate that CPV-2 infection triggers the host translation shutoff, a process in which the nonstructural protein 1 (NS1) plays a pivotal role. Our findings indicate that the CPV-2 NS1-induced host translation shutoff is not associated with transcription, protein degradation pathways, or eIFα phosphorylation, but rather involves the reduction of phosphorylation of the mammalian target of rapamycin (mTOR). In conclusion, this research reveals that CPV-2 NS1 induces a host translation shutoff by reducing mTOR phosphorylation, a mechanism that could potentially inform the development of more efficacious control and therapeutic strategies for CPV-2 and other parvoviral infections.

**IMPORTANCE:**

Autonomous parvoviruses, which possess compact genomes, are obligate intracellular parasites that necessitate host cell division for their replication cycle. Consequently, the modulation of host translation and usurpation of cellular machinery are hypothesized to facilitate immune evasion, enhance viral transmission, and perpetuate long-term infection. Despite the biological significance, the precise mechanisms by which autonomous parvoviruses regulate host translation remain understudied. Our study elucidates that CPV-2 infection induces a shutoff of host translation through the attenuation of mTOR phosphorylation. This mechanism may enable the virus to subvert the host immune response and engender pathogenic effects.

## INTRODUCTION

The interplay between viruses and their hosts is an intricate and highly regulated process. Viral manipulation of host translation machinery results in the global suppression of protein synthesis within the host cell (1), a strategy that not only aids the virus in evading the immune response but also enables the diversion of host resources for the synthesis of viral proteins.

Translation is a meticulously regulated cellular process. The general cessation of translation, or global translation arrest, is typically induced by the phosphorylation of the eukaryotic translation initiation factor 2α (eIF2α) (2). Additionally, the mTORC1-S6K signaling pathway exerts a significant influence over the regulation of translation initiation (3). The initiation rate of translation is primarily governed by the recognition of the 5′ cap by the eIF4F complex, which consists of eIF4G, eIF4A, and eIF4E. The binding of eIF4F to the cap structure is subject to regulation by eIF4E binding proteins (4E-BPs). mTORC1 counteracts this inhibition through the phosphorylation of 4E-BPs, thereby promoting translation initiation (4). Furthermore, mTORC1 phosphorylates S6 kinase, which subsequently phosphorylates a plethora of substrates that are integral to the translational process (5). Beyond the mTORC1 pathway, the RAS-MAPK signaling cascade also serves as a pivotal regulator of translation, being responsible for the phosphorylation of eIF4B and eIF4E through the action of MNK kinases (3, 6).

Influenza A virus (IAV) ensures the efficacious translation of its viral mRNAs through the implementation of host shutoff mechanisms and a “cap-snatching” strategy (7, 8). Porcine reproductive and respiratory syndrome virus (PRRSV) mediates the shutdown of host translation by inducing the phosphorylation of eIF2α and inhibiting the mTOR signaling pathway (9). The translational shutoff observed in Newcastle disease virus (NDV) infections is attributed to the sustained phosphorylation of eIF2α (10). Similarly, the pE66L protein of African swine fever virus (ASFV) suppresses host translation by promoting eIF2α phosphorylation (11). The 3C proteases of poliovirus, calicivirus, seneca Valley virus, and duck hepatitis A virus are known to cleave the poly(A)-binding protein, thereby interfering the cellular protein translation (12–15). Collectively, these mechanisms underscore the host translation system as a significant target for viral manipulation.

Parvoviruses constitute a class of small DNA viruses, the majority of which are dependent on host cell division for replication and are entirely reliant on the host translational machinery for the synthesis of viral proteins (16). Infection by parvoviruses modulates various cellular processes, including the host’s innate immune responses (17), apoptosis (18), cell cycle arrest (19), autophagy (20), and DNA damage response (21). A previous study demonstrated that the nonstructural (NS) proteins of Junonia coenia densovirus (JcDV) commandeer the cellular translational machinery, favoring the translation of viral mRNAs through the modulation of the mTOR signaling pathway, and ultimately mediating autophagy (22). The NS1 protein of the minute virus of mice (MVM) induces cell cycle arrest in the S/G2 phase, exerts cytotoxic effects, and thereby influences the synthesis and phosphorylation of host proteins (19). Additionally, it exhibits a selective targeting of the host cell’s cytoskeletal components (23). Infection with human parvovirus B19 has also been associated with abnormal mitosis (24). These findings collectively illustrate the different mechanisms by which parvoviruses regulate host signal pathway during infection.

Canine parvovirus type 2 (CPV-2) is a quintessential member of the Parvoviruses family, exhibiting significant pathogenicity to canine and feline hosts. The CPV-2 genome is organized into two coding regions: the 3′ region, which encodes the nonstructural proteins NS1 and NS2, and the 5′ region, which encodes the structural proteins VP1 and VP2 (25). Mature virions also contain VP3, a product derived from the proteolytic cleavage of VP2. As a phosphorylated nucleo-cytoplasmic shuttling protein (26), the NS1 protein of CPV-2 is implicated in multiple processes, including the regulation of viral genome replication (27), the mediation of DNA damage responses (21), the induction of apoptosis (18), and the arrest of cell cycle (27). Furthermore, it has been reported to exert oncolytic effects (28). Given its status as a primary virulence factor, elucidating the intricate interactions between NS1 and the host during CPV-2 infection is crucial for the advancement of more efficacious control and therapeutic strategies. However, the mechanisms governing host translation regulation during CPV-2 infection remain largely obscure. In this study, we concentrate on CPV-2 and demonstrate that the NS1 protein induces a shutoff of host translation, specifically by inducing a reduction of mTOR phosphorylation, a key event in the translational shutoff process.

## RESULTS

### CPV-2 infection induces protein expression shutoff in cells

**Fig 1:**
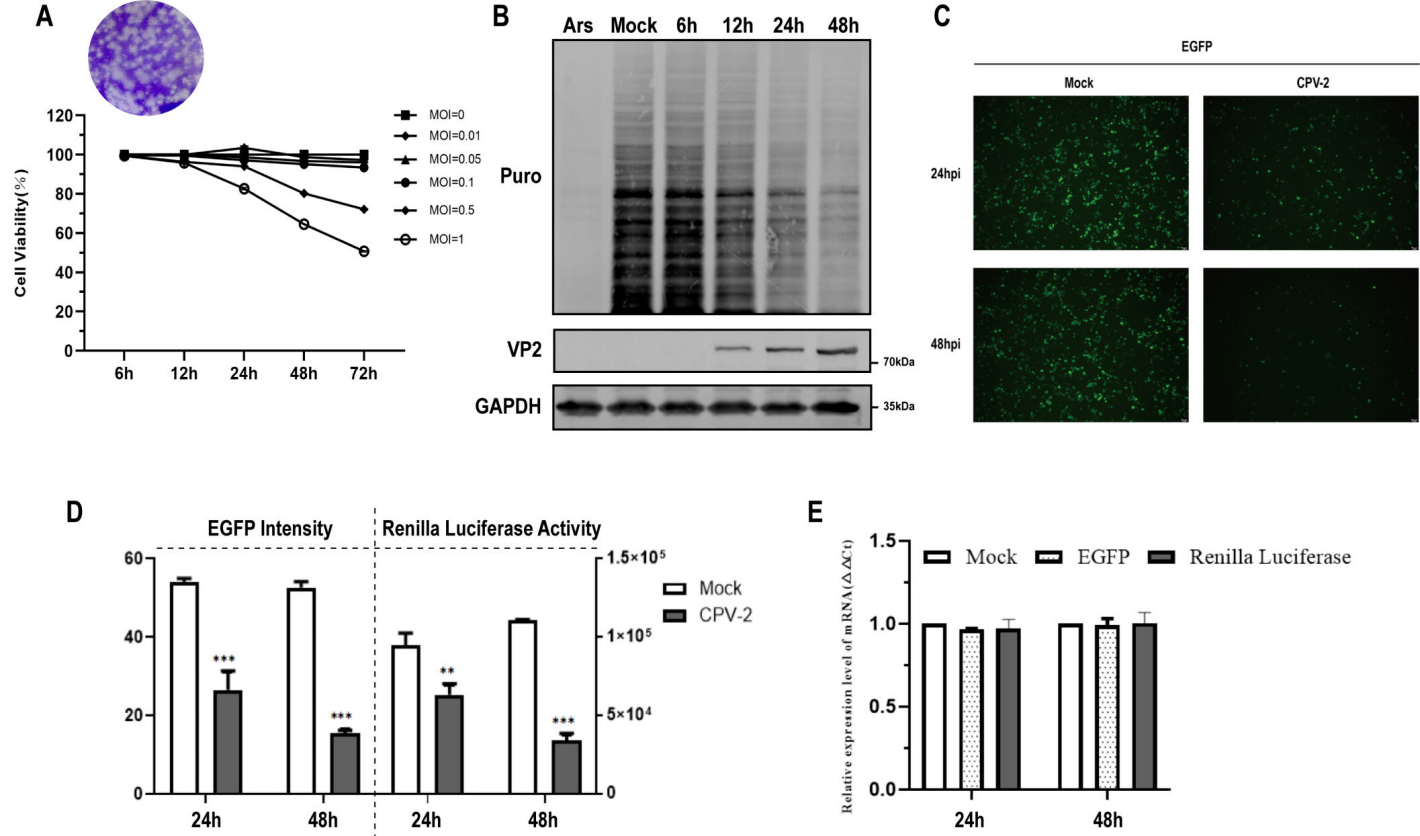
CPV-2 infection induces protein expression shutoff in cells. (**A**) CPV-2 replication in MDCK cells was quantified using a plaque assay on F81 cells, and the cytotoxicity of CPV-2 infection was assessed with the CCK-8. (**B**) MDCK cells, cultured in 12-well plates, were infected with CPV-2 at an MOI of 0.1 for 6, 12, 24, or 48 h. Uninfected cells treated with 0.5 mM Ars for 1 h served as a positive control. Western blot analysis was performed after cell lysis. Nascent polypeptides labeled with puromycin were detected using an anti-puromycin monoclonal antibody. An anti-VP2 antibody was utilized to verify CPV-2 infection, and an anti-GAPDH antibody served as a loading control. (**C, D**) MDCK cells, cultured in 6-well plates, were transfected with 4 µg of either pEGFP-C3 or pRL-TK plasmid for 24 h, followed by inoculation with CPV-2 at a MOI of 0.1. EGFP fluorescence was observed and captured using a fluorescence microscope at 24 and 48 hpi (**C**). The fluorescence intensity of EGFP and the activity of Renilla luciferase were quantified (**D**), with results presented as means ± standard deviation (SD) from three independent experiments. (**E**) qPCR assays were conducted to determine the relative mRNA expression levels of EGFP and Renilla luciferase following CPV-2 infection at 24 or 48 hpi. The results are presented as means ± SD from three independent experiments.

CPV-2 induces the formation of plaques on F81 cells, thereby facilitating the assessment of viral titer through the use of these cells. Furthermore, the cytotoxic effects of CPV-2 infection on MDCK cells were ascertained utilizing the Cell Counting Kit-8 (CCK-8) assay. It was observed that the cell viability remains at 100% when the multiplicity of infection (MOI) is ≤0.1 for CPV-2 (Fig. 1A). Consequently, an MOI of 0.1 was adopted for CPV-2 in all subsequent experimental procedures involving infected cells.

To evaluate the effects of CPV-2 on the protein expression machinery, a short pulse with puromycin, an aminoacyl-tRNA analog that is incorporated into nascent polypeptides (29), was utilized to assess protein synthesis. The expression of host proteins was similarly found to be suppressed during CPV-2 infection at both 24 and 48 hpi, with the most pronounced inhibition at 48 h post inoculation (hpi) (Fig. 1B). Subsequently, MDCK cells were transfected with either pEGFP-C3 or pRL-TK plasmids, followed by inoculation with CPV-2. Both the fluorescence intensity of EGFP (Fig. 1C and D) and the activity of Renilla luciferase (Fig. 1D) were observed to decrease at 24 and 48 hpi, suggesting a downregulation of heterologous protein expression due to CPV-2 infection. Total RNA was extracted from the samples, and the levels of EGFP and Renilla luciferase mRNA were measured using quantitative real-time PCR (qPCR) analysis. Notably, no significant differences were detected in the mRNA levels of EGFP or Renilla luciferase following CPV-2 infection (Fig. 1E). These findings collectively indicate that CPV-2 infection induces a heterologous protein expression shutoff that is independent of mRNA transcription.

### NS1 induce host protein expression shutoff

**Fig 2:**
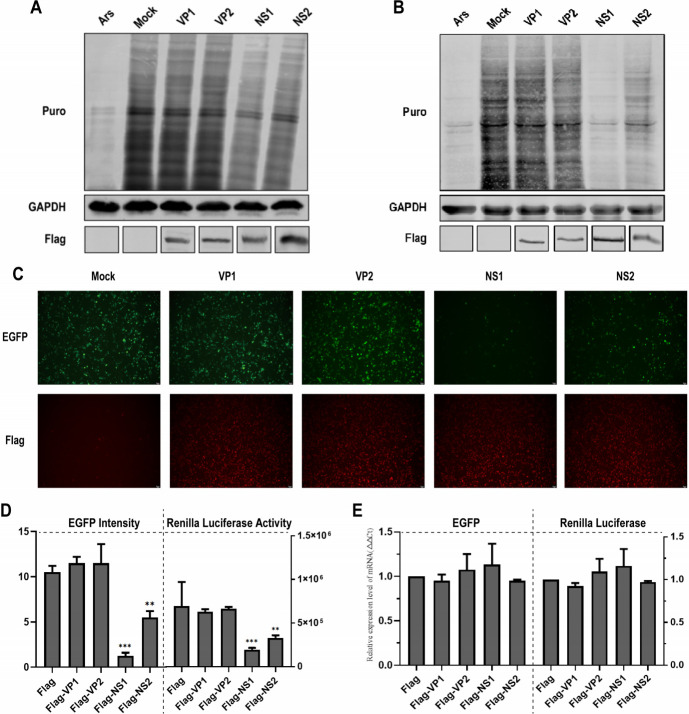
Effects of viral proteins on host protein expression. (**A and B**) MDCK cells (**A**) or HEK293T cells (**B**), cultured in 12-well plates, were transfected with 1 µg of plasmids encoding Flag-VP1, Flag-VP2, Flag-NS1, and Flag-NS2 for 48 h. Following treatment with puromycin, translation was measured using the method described in the text. (**C and D**) HEK293T cells, cultured in 6-well plates, were transfected with 4 µg of pEGFP-C3 or pRL-TK plasmid, along with co-transfection of plasmids encoding for the viral proteins Flag-VP1, Flag-VP2, Flag-NS1, and Flag-NS2. At 48 hpt, EGFP fluorescence was observed and captured using a fluorescence microscope (**C**). The intensity of EGFP fluorescence and the activity of Renilla luciferase were quantified (**D**), with results presented as means ± SD from three independent experiments. (**E**) qPCR assays were conducted to determine the relative mRNA expression levels of EGFP and Renilla luciferase after transfection with different viral protein-encoding plasmids for 48 h. Results are displayed as means ± SD from three independent experiments.

To identify the viral protein responsible for the shutoff of host protein expression, we co-transfected MDCK cells or HEK293T cells with plasmids encoding for each of the viral proteins (Flag-VP1, Flag-VP2, Flag-NS1, and Flag-NS2), the ribopuromycylation assay demonstrated a significant decrease in the synthesis of nascent polypeptides following the introduction of NS1 or NS2 proteins in MDCK cells (Fig. 2A) and HEK 293T cells (Fig. 2B). Furthermore, we co-transfected or HEK293T cells with plasmids encoding for each of the viral proteins along with either pEGFP-C3 or pRL-TK. The expression of Flag-VP1, Flag-VP2, Flag-NS1, and Flag-NS2 was confirmed using an immunofluorescence assay (IFA) at 48 h post-transfection (hpt) (Fig. 2C). In the groups transfected with VP1 or VP2, the intensity of EGFP fluorescence was comparable to that of the control group (Fig. 2C and D). In contrast, a significant reduction in EGFP fluorescence intensity was observed in the groups transfected with NS1 or NS2 (Fig. 2C and D). Consistently, the activity of Renilla luciferase was also found to be inhibited by the presence of NS1 or NS2 (Fig. 2D). Additionally, total RNA was extracted, and the levels of EGFP and Renilla luciferase mRNA were quantified using qPCR analysis. Compared to the empty vector transfection, no significant differences were noted in the mRNA levels of EGFP or Renilla luciferase in cells transfected with Flag-VP1, Flag-VP2, Flag-NS1, and Flag-NS2, respectively (Fig. 2E).

These findings suggest that both the NS1 and NS2 proteins are implicated in the shutoff of host protein expression, with NS1 exhibiting the most potent inhibitory effect (Fig. 2A through D). Consequently, the NS1-induced host protein expression shutoff induced by CPV-2 was selected for further investigation to elucidate the underlying mechanisms.

### The CPV-2 NS1-induced shutoff of host protein expression is independent of transcriptional regulation, protein degradation pathways, and eIF2α phosphorylation

No significant differences were observed in the levels of EGFP or Renilla luciferase mRNA in cells following CPV-2 infection (Fig. 1D) or transfection with CPV-2 NS1 (Fig. 2D). This finding indicates that the shutoff of host protein expression induced by CPV-2 NS1 is independent of transcriptional regulation.

**Fig 3:**
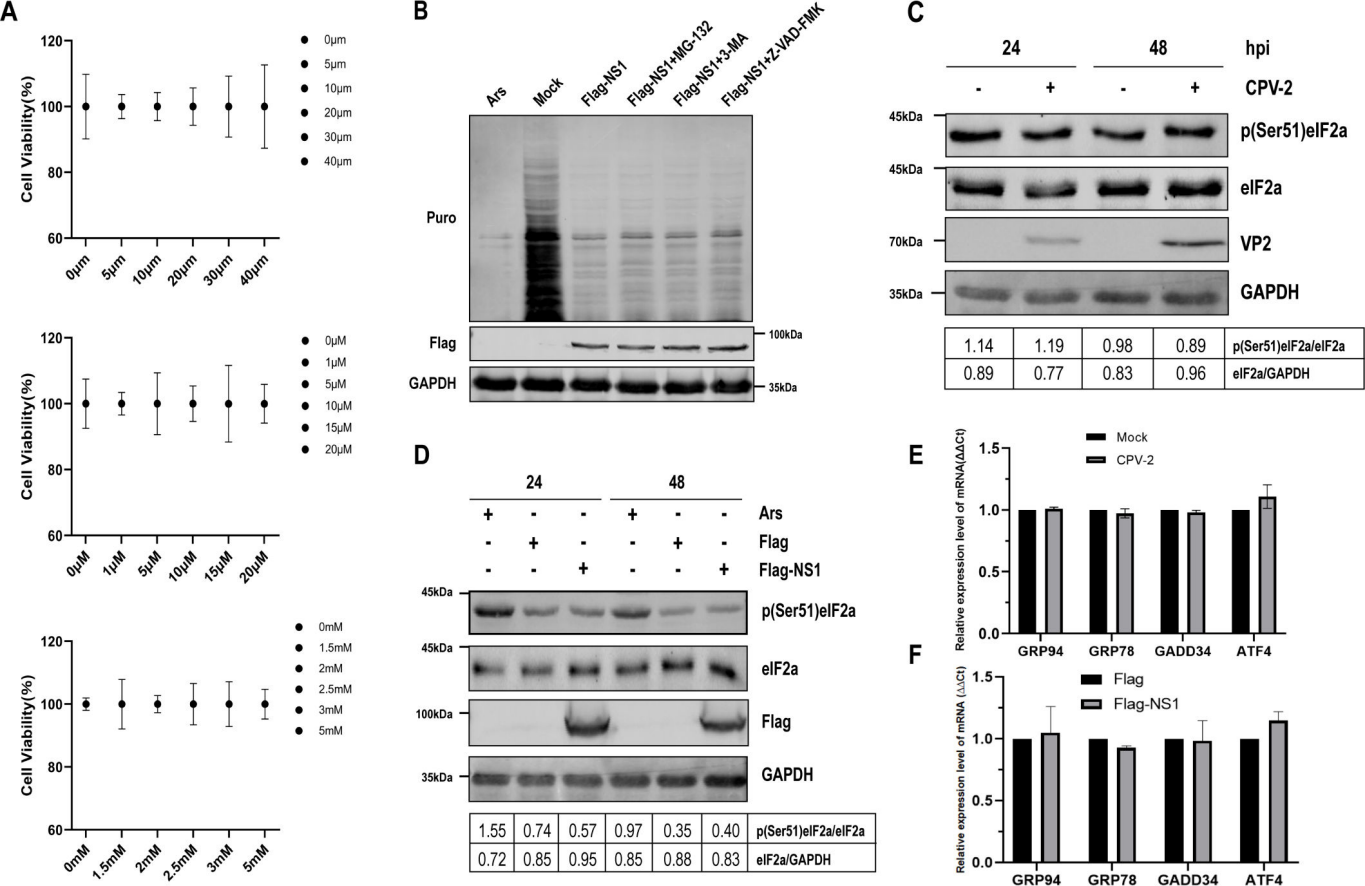
NS1-induced host protein translation shutoff is independent of protein degradation pathways and eIF2α phosphorylation. (**A**) HEK293T cells were seeded in 96-well plates, and viability was assessed after treatment with MG-132, 3-MA, and Z-VAD-FMK using the CCK-8 assay. (**B**) HEK293T cells, cultured in 12-well plates, were transfected with 1 µg of Flag-NS1 or an empty vector control. Sixteen hours post-transfection, the cells were treated with MG-132 (20 µM), 3-MA (5 mM), or Z-VAD-FMK (10 µM) for an additional 32 h. Subsequent to puromycin treatment, translation was measured as previously described. (**C**) MDCK cells, cultured in 12-well plates, were infected with CPV-2 at an MOI of 0.1. At 24 and 48 hpi, cells were harvested, and the expression levels of eIF2α and phosphorylated eIF2α were analyzed by Western blotting. An anti-VP2 antibody was utilized to verify CPV-2 infection, while an anti-GAPDH antibody served as a loading control. The phosphorylated protein bands were quantified using ImageJ analysis, with grayscale values normalized to the protein-to-GAPDH ratio. (**D**) HEK293T cells, cultured in 12-well plates, were transfected with 1 µg of Flag-NS1. The cells were harvested and analyzed by Western blotting as described in panel C. An anti-Flag antibody was used to confirm NS1 expression. (**E and F**) MDCK cells (**E**) and HEK293T cells (**F**), cultured in 12-well plates, were transfected with 1 µg of Flag-NS1 plasmid or an empty vector control. At 48 hpt, total RNA was extracted and analyzed for the expression of endogenous GRP94, GRP78, GADD34, and ATF4 mRNAs using qPCR. Results are presented as means ± SD from three independent experiments.

Host protein expression is intricately regulated through multiple pathways. The ubiquitin-proteasome system, autophagy-lysosome pathway, and apoptosis are recognized as the three principal intracellular protein degradation pathways in eukaryotic cells (9, 30, 31). To evaluate the potential involvement of these pathways in the NS1-induced shutoff of host translation, HEK293T cells transfected with NS1 were treated with the proteasomal inhibitor MG132, the autophagy inhibitor 3-MA, or the apoptosis inhibitor Z-VAD-FMK. Despite these treatments, cellular translation levels showed no significant recovery (Fig. 3B). From these observations, we conclude that the NS1-induced shutoff of host translation is not associated with the proteasomal, autophagic, or apoptotic pathways.

The phosphorylation of eIF2α is a pivotal mechanism in the regulation of translation control (32, 33). Both NDV infection and ASFV pE66L-induced translation shutoff have been attributed to the activation of eIF2α phosphorylation, with the eIF2α pathway being among the first to be activated in the endoplasmic reticulum stress (ERS) response (10, 11). Consequently, we explored the potential involvement of the eIF2α pathway in NS1-induced translation shutoff. To ascertain the role of eIF2α in NS1-induced translational arrest, we quantified the levels of eIF2α phosphorylation in cells following CPV-2 infection or transfection with CPV-2 NS1. Our results indicated that, whereas Ars treatment (Fig. 3D) led to a significant increase in eIF2α phosphorylation, neither CPV-2 infection (Fig. 3C) nor NS1 transfection (Fig. 3D) induced such an effect.

As indicators of ERS, we initially assessed the mRNA expression levels of glucose-regulated protein 94 (GRP94) and glucose-regulated protein 78 (GRP78) following CPV-2 infection or NS1 expression. The findings demonstrated that neither GRP94 nor GRP78 mRNA levels were upregulated following CPV-2 infection (Fig. 3E) or NS1 expression (Fig. 3F). Phosphorylation of eIF2α is known to significantly increase the mRNA levels of growth arrest and DNA damage-inducible gene 34 (GADD34) and activating transcription factor 4 (ATF4) (34). Ultimately, we observed no upregulation in the transcriptional levels of GADD34 or ATF4 in cells following CPV-2 infection (Fig. 3E) or NS1 expression (Fig. 3F). These findings indicate that CPV-2 NS1 does not induce eIF2α phosphorylation, suggesting that the eIF2α pathway is not implicated in the NS1-induced host translation shutoff.

### NS1 expression and CPV-2 infection negatively regulate the mTOR signaling pathway

**Fig 4:**
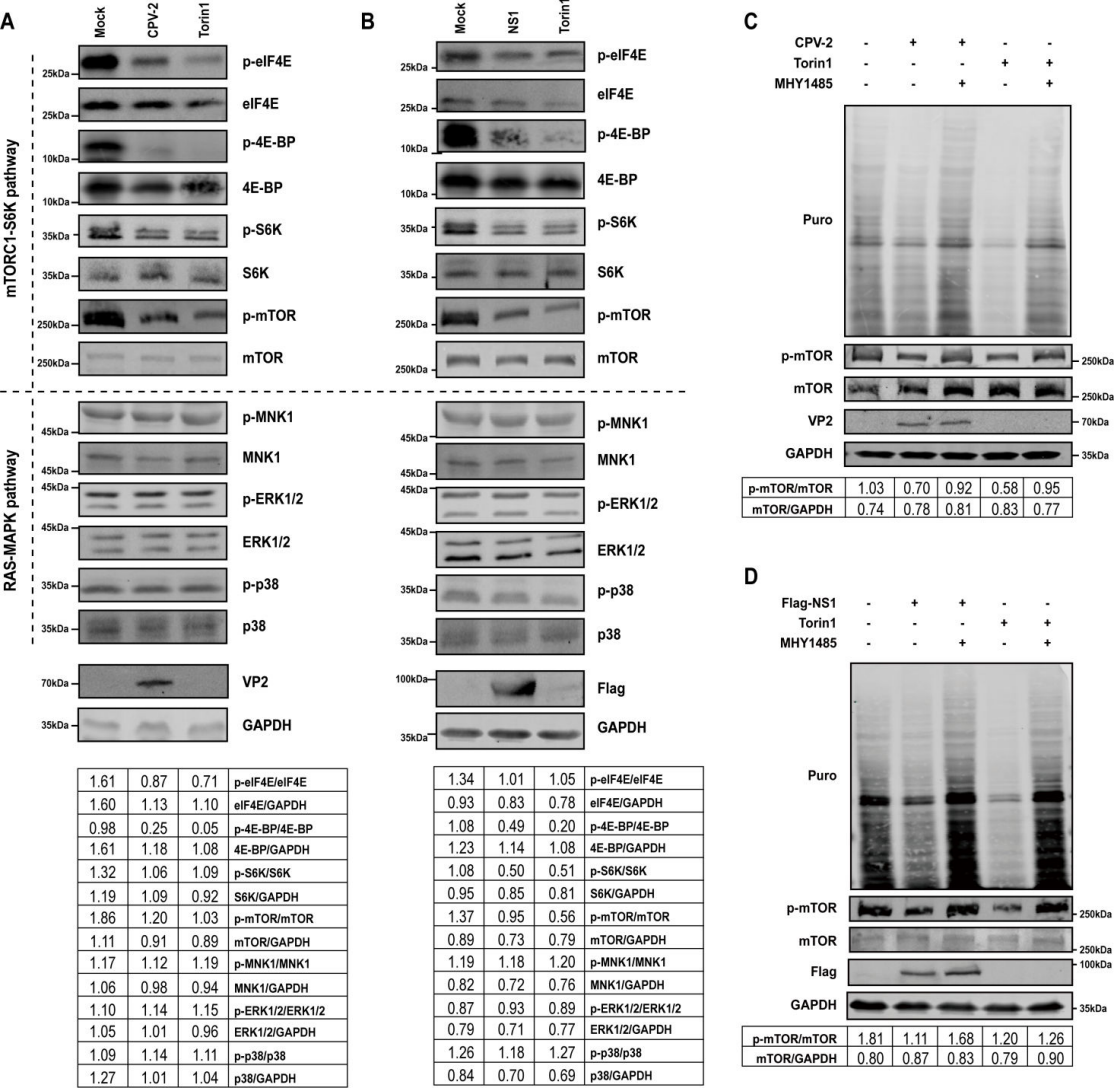
mTOR signaling pathway is attenuated by CPV-2 infection and NS1 overexpression. (**A**) MDCK cells, cultured in 12-well plates, were infected with CPV-2 at an MOI of 0.1. Cells treated with Torin1 (250 nM for 6 h) and harvested at 48 hpi, analyzed by Western blotting using the same methodology as described in panel A. (**B**) HEK293T cells, cultured in 12-well plates, were transfected with 1 µg of Flag-NS1. Cells treated with Torin1 (250 nM for 6 h) served as a positive control for the inhibition of translation. Harvested at 48 hpt, cell lysates were subjected to immunoblotting with antibodies specific to the indicated proteins. Quantification of phosphorylated proteins was performed using ImageJ, with grayscale values normalized to the respective protein-to-GAPDH ratios. (**C**) MDCK cells, cultured in 12-well plates, were infected with CPV-2 at an MOI of 0.1. At 48 hpi, the cells were treated and analyzed by Western blotting using the same methodology as described in panel C. (**D**) HEK293T cells, cultured in 12-well plates, were transfected with 1 µg of Flag-NS1. At 48 hpt, the cells were treated with MHY1485 (2 µM for 1 h) served as an mTOR pathway activator, followed by a 25-min incubation with puromycin. Western blotting was employed to assess the levels of translation and mTOR phosphorylation.

In eukaryotic cells, mRNA translation is predominantly cap-dependent, necessitating the binding of eIF4E to the methylated cap structure (35). The activity of eIF4E is modulated by the mTORC1-S6K and RAS-MAPK signaling pathways (9). Given this, we proceeded to investigate the potential impairment of eIF4E phosphorylation or its regulatory mechanisms by CPV-2. To accomplish this, MDCK cells were infected with CPV-2, and the expression levels of total eIF4E, 4E-BP1, p70-S6K (S6K), mTOR, MNK1, ERK1/2, and p38, as well as their phosphorylation states, were assessed using Western blotting. As a positive control, cells were exposed to Torin1, an mTOR inhibitor known to impede mTOR phosphorylation, thereby reducing the activation of downstream effectors (36). Our findings revealed that CPV-2 infection significantly reduced the phosphorylation of mTOR, 4E-BP1, S6K, and eIF4E, while the phosphorylation levels of MNK1 and p38 remained unaffected (Fig. 4A). Furthermore, We examined the impact of NS1 expression on the mTORC1-S6K and RAS-MAPK signaling pathways in HEK293T cells. The outcomes were in concordance with our hypothesis, indicating that NS1 expression exerts a negative regulatory effect on the mTOR signaling pathway (Fig. 4B).

To further elucidate the impact of mTOR signaling pathway activation on translation in CPV-2-infected cells, we employed the mTOR pathway activator MHY1485 at a concentration of 2 µM (9, 37). MHY1485 effectively reversed the translational shutoff induced by Torin1. Furthermore, MHY1485 significantly rescued mTOR phosphorylation and restored translation levels in CPV-2-infected cells (Fig. 4C) and NS1-expressing cells (Fig. 4D). These observations underpin our conclusion that CPV-2 inhibits host cell translation by suppressing the mTOR signaling pathway.

The results collectively demonstrate that both CPV-2 infection and NS1 expression downregulate the mTOR signaling pathway, which may account for the CPV-2 NS1-induced host translation shutoff.

## DISCUSSION

Elucidating the intricate interactions between viruses and host cells is crucial for understanding the host’s antiviral responses and the mechanisms by which viruses evade host clearance. In this context, the viral inhibition of host cell translation represents a significant strategy to facilitate viral replication while evading immune surveillance. The literature has documented various mechanisms through which viruses can induce translation shutoff in host cells (7–9, 11). Our study contributes to this body of knowledge by demonstrating that the NS1 protein of CPV-2 suppresses the mTOR signaling pathway, thereby attenuating host translation. In summary, the inhibitory effect of NS1 on host cell translation may enhance our comprehension of virus-induced pathogenicity. Prior research has established that NS1 is involved in regulating viral genome replication (38), inducing apoptosis (18), causing cell cycle arrest, inducing DNA damage (21), and exerting oncolytic effects (28). These findings underscore the multifunctional nature of the NS1 protein. In this study, we highlight the role of CPV-2 NS1 in mediating the shutoff of host translation, affirming its status as a major virulence factor.

In the mechanism of CPV-2-induced translation shutoff, the NS1 protein exerts the most significant influence. While viruses are known to disrupt cap-dependent host translation, they may concurrently facilitate their own protein synthesis through alternative pathways (39). Despite the presence of poly(A) structures within the CPV-2 genome (16, 25), viral proteins are synthesized with high efficiency. As an early viral protein, NS1 is transcribed and translated preferentially. It initially appears in the host cell following CPV-2 infection and subsequently becomes distributed throughout the nucleus (26). With the advancement of the infection, there is a substantial accumulation of NS1 mRNA and protein, leading to the displacement of host chromatin (40, 41), a phenomenon that may enhance the preferential nuclear export of viral mRNAs.

Virus-infected cells typically elicit a stress response, wherein the phosphorylation of eIF2α results in the attenuation of host translation (9). Nonetheless, our study observed that CPV-2 infection and expression of the NS1 protein did not utilize this pathway. The levels of GRP94, GRP78, GADD34, and ATF4 were not upregulated, suggesting that the virus did not induce ERS. Notably, CPV-2 was found to inhibit the mTOR signaling pathway, consequently reducing the phosphorylation of eIF4E and reducing host translation levels. This mode of action bears resemblance to the mechanism employed by JcDV (22).

Infection with CPV-2 targets rapidly dividing enterocytes within the intestinal epithelial crypts of dogs or cats, resulting in hemorrhagic diarrhea (42, 43). Additionally, CPV-2 infection of the persistently dividing cardiomyocytes in puppies can lead to myocarditis (44). Furthermore, the rapid fetal development and growth, which necessitate the synthesis of substantial amounts of protein, create an environment conducive to viral replication. However, in the event of CPV-2 infection during this critical period, the host’s protein synthesis machinery may be commandeered for viral production. This manipulation can disrupt the antiviral immune response and impede cell division, ultimately culminating in the manifestation of clinical symptoms.

The shutoff of host translation due to CPV-2 infection impedes the normal execution of the host’s antiviral response, an effect analogous to that observed with the B19-NS1 protein (17). Consequently, the development of effective preventative and therapeutic strategies for CPV-2 infection is of paramount importance. Vaccination currently represents the primary preventative measure; however, existing commercial vaccines do not entirely safeguard against CPV-2 variants, with the CPV-2c variant being increasingly prevalent in various regions (45, 46). Thus, there is an imperative need to explore alternative preventative and treatment approaches. Targeting the mTOR signaling pathway may offer a promising avenue, given the relative conservation of the core proteins within this pathway.

**Fig 5:**
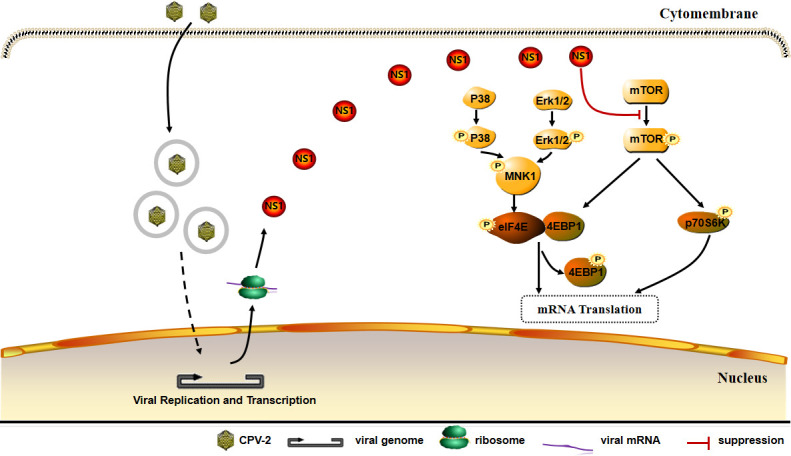
Schematic diagram of the mechanism of CPV-2 NS1-induced host translation shutoff.

In summary (as depicted in Fig. 5), the expression of the NS1 protein in cells infected with CPV-2 leads to a reduction in the phosphorylation of mTOR and its downstream effectors, 4E-BP1 and S6K, thereby inducing a shutoff of host translation.

## MATERIALS AND METHODS

### Cells and viruses

MDCK cells and HEK 293T cells were obtained from iCell Bioscience (Shanghai, China) and cultured in Dulbecco’s modified Eagle’s medium (DMEM) supplemented with 10% Fetal Bovine Serum (FBS) and a 1 × mixture of penicillin (100 U/mL) and streptomycin (10 µg/mL). The cells were maintained in a humidified incubator with 5% CO2 at 37°C. The CPV-2 strain, with GenBank accession number OP957419, was isolated during our etiological investigations.

### Plasmids and transfection

The pRL-TK, pEGFP-C3, and p3×Flag-CMV10 plasmids were maintained in our laboratory. The CPV-2 genes encoding VP2, VP1, and NS1 (with five nucleotide mutations introduced at the mRNA splicing site to prevent the expression of NS2), as well as NS2, were synthesized and cloned into the p3×Flag-CMV10 vector. All plasmids constructed in this study underwent sequencing to ensure the absence of unintended mutations.

For transfection, cells were plated in culture plates and transfected with Lipofectamine 3000 (Thermo Fisher Scientific, MA, USA) according to the manufacturer’s protocol, using the respective plasmids once the cells reached 70%–80% confluence.

### Chemicals and reagents

Puromycin, MG132 (a proteasome inhibitor), 3-MA (an autophagy inhibitor), and Z-VAD-FMK (an apoptosis inhibitor) were procured from Meilunbio Co. (Dalian, China). Torin 1 was sourced from Medchem Express (Monmouth Junction, NJ, USA). Sodium arsenite (Ars) and MHY1485 were acquired from Sigma-Aldrich (USA). The CCK-8 assay was purchased from Beyotime, Inc.

### Antibodies

A mouse monoclonal antibody against puromycin was sourced from Millipore (Billerica, MA, USA). The mouse monoclonal anti-CPV-VP2 antibody was obtained from Biocare Diagnostics Ltd. (Zhuhai, Guangdong, China). The mouse monoclonal anti-FLAG M2 antibody was purchased from Sigma-Aldrich (St. Louis, MO, USA).

Rabbit monoclonal antibodies against p-MNK1, eIF2α, p-eIF2α, p38, p-p38, ERK1/2, and p-ERK1/2 were procured from Cell Signaling Technology (Beverly, MA, USA). Rabbit monoclonal antibodies against mTOR, p-mTOR, 4E-BP1, p-4E-BP1, eIF4E, p-eIF4E, S6K, and p-S6K were obtained from ZEN-BIOSCIENCE (Chengdu, Sichuan, China). The IRDye 800CW Goat (polyclonal) Anti-Rabbit/Mouse IgG (H + L) secondary antibody was purchased from LI-COR.

### Viral titer

The replication of CPV-2 in cells was assessed by plaque assay on F81 cells. Seed 2 × 10^5^ cells per well in a 48-well plate. Dilute the virus to concentrations ranging from 10^−1^ to 10^−8^ and inoculate onto the plates. Overlay each well with Avicel. After a 3-day incubation, aspirate the Avicel, stain the cell monolayer with Crystal Violet, remove the stain, and enumerate plaques. Calculate the virus titer (pfu/mL) using the formula: Virus titre (pfu/mL) = (number of plaques × 10 × dilution factor).

### Cytotoxicity assay

Cytotoxicity of CPV-2 infection was determined using the CCK-8. Seed MDCK cells in 96-well plates and infect with CPV-2 at various MOI. Perform experiments in triplicate, including a blank control. After incubation for 24, 48, or 72 h, add the Cell Counting Kit-8 solution and incubate for 1 h. Measure absorbance at 450 nm. Calculate cytotoxicity as: 1 − (sample absorbance/blank absorbance).

### EGFP fluorescence and luciferase reporter assay

MDCK and HEK293T cells were seeded in 6- or 12-well plates for CPV-2 infection or plasmid transfection. Transfect 4 µg (for 6-well plates) or 1 µg (for 12-well plates) of pEGFP-C3 or pRL-TK plasmid into cells for 24 h, followed by CPV-2 inoculation (MOI = 0.1), or co-transfect with viral protein plasmids. After 24 or 48 h, observe EGFP fluorescence intensity using a fluorescence microscope (Leica, Germany). Capture images with set exposure and gain. Measure EGRP fluorescence intensity using ImageJ (47), which represents protein expression levels. Determine Renilla luciferase activity by a method described previously (48). Present data as mean values ± SD from three independent experiments.

### Ribopuromycylation assay

Monitor cellular peptide synthesis to assess the effect of CPV-2 or viral proteins on translation. Seed MDCK/HEK293T cells in 6- or 12-well glass bottom plates for CPV-2 infection or plasmid transfection. After infection (MOI = 0.1) or transfection with viral protein plasmids for 24 or 48 h, add puromycin (5 µg/mL) to the culture medium and incubate for 25 min at 37°C. Wash cells with PBS, extract proteins with lysis buffer, and analyze by Western blotting. Use an anti-puromycin monoclonal antibody to detect puromycin-labeled nascent polypeptides. The methodology follows previous studies (49, 50).

### Quantitative Real-Time PCR

Extract total RNA using an RNA Rapid Extraction Kit (Fastagen, China) and reverse transcribe into cDNA using a PrimeScript RT reagent kit (Takara, Beijing, China). Perform qPCR with the synthesized cDNA and SYBR master mix (GenStar, Beijing, China). Normalize gene expression to GAPDH and calculate relative mRNA levels using the ΔΔCt method. Conduct qPCR on a Light Cycler 480 qPCR instrument (Roche). List qPCR primers: GAPDH (F/R: CAAGAAGGTAGTGAAGCAGGCATC/TCGAAGGTGGAAGAGTGGGTG), GRP94 (F/R: TGGGCAGTTTGGTGTCG/GTTCCCTCGTGGGTCAG), GRP78 (F/R: GAAACTGCGGAGGCTTAT/CCAGGTCAAACACCAGGATA), GADD34 (F/R:AAAGGTCCGTTTCTCCG/CCCAACATGGCTCAAGG), ATF4 (F/R: CGAATGGCTGGCTTTG/GCTCATCTGGCATGGTTT), EGFP (F/R:AAGGGCATCGACTTCAAGGA/CTTCTCGTTGGGGTCTTGC),TK (F/R:TCATGGCCTCGTGAAATCCC/TGGCACCTTCAACAATAGCAT).

### Western blotting

Wash MDCK/HEK293T cells with precooled PBS and lyse in a buffer containing phosphatase inhibitors and PMSF. Resolve extracted proteins by 4%–20% SDS-PAGE and transfer to polyvinylidene difluoride membranes (Millipore, USA). Block membranes with 5% skim milk, incubate with primary antibodies overnight at 4°C, then with IRDye 800CW conjugated anti-rabbit/mouse IgG secondary antibody for 30 min. Analyze membranes using an Odyssey infrared imaging system (LI-COR, USA).

### Statistical analyses

All data represent at least two independent experiments, wherein measurements were performed in triplicate. Data were analyzed by two-tailed Student’s *t* test using GraphPad Prism, version 8. *P* values of < 0.05 were considered statistically significant. The data in the figure are presented as the mean ± SD of three independent experiments. **P < 0.01, ***P < 0.001, ****P < 0.0001.

## Data Availability

The data are all displayed in this manuscript.
